# Wnt, RSPO and Hippo Signalling in the Intestine and Intestinal Stem Cells

**DOI:** 10.3390/genes9010020

**Published:** 2018-01-08

**Authors:** Vitezslav Kriz, Vladimir Korinek

**Affiliations:** Department of Cell and Developmental Biology, Institute of Molecular Genetics of the CAS, v. v. i., Videnska 1083, 142 20 Prague 4, Czech Republic; krizv@img.cas.cz

**Keywords:** colorectal cancer, Hippo pathway, LGR, R-Spondins, YAP1/TAZ, Wnt/β-catenin signalling

## Abstract

In this review, we address aspects of Wnt, R-Spondin (RSPO) and Hippo signalling, in both healthy and transformed intestinal epithelium. In intestinal stem cells (ISCs), the Wnt pathway is essential for intestinal crypt formation and renewal, whereas RSPO-mediated signalling mainly affects ISC numbers. In human colorectal cancer (CRC), aberrant Wnt signalling is the driving mechanism initiating this type of neoplasia. The signalling role of the RSPO-binding transmembrane proteins, the leucine-rich-repeat-containing G-protein-coupled receptors (LGRs), is possibly more pleiotropic and not only limited to the enhancement of Wnt signalling. There is growing evidence for multiple crosstalk between Hippo and Wnt/β-catenin signalling. In the *ON* state, Hippo signalling results in serine/threonine phosphorylation of Yes-associated protein (YAP1) and tafazzin (TAZ), promoting formation of the β-catenin destruction complex. In contrast, YAP1 or TAZ dephosphorylation (and YAP1 methylation) results in β-catenin destruction complex deactivation and β-catenin nuclear localization. In the Hippo *OFF* state, YAP1 and TAZ are engaged with the nuclear β-catenin and participate in the β-catenin-dependent transcription program. Interestingly, YAP1/TAZ are dispensable for intestinal homeostasis; however, upon Wnt pathway hyperactivation, the proteins together with TEA domain (TEAD) transcription factors drive the transcriptional program essential for intestinal cell transformation. In addition, in many CRC cells, YAP1 phosphorylation by YES proto-oncogene 1 tyrosine kinase (YES1) leads to the formation of a transcriptional complex that includes YAP1, β-catenin and T-box 5 (TBX5) DNA-binding protein. YAP1/β-catenin/T-box 5-mediated transcription is necessary for CRC cell proliferation and survival. Interestingly, dishevelled (DVL) appears to be an important mediator involved in both Wnt and Hippo (YAP1/TAZ) signalling and some of the DVL functions were assigned to the nuclear DVL pool. Wnt ligands can trigger alternative signalling that directly involves some of the Hippo pathway components such as YAP1, TAZ and TEADs. By upregulating Wnt pathway agonists, the alternative Wnt signalling can inhibit the canonical Wnt pathway activity.

## 1. Introduction

In this review, we describe the molecular mechanisms and possible crosstalk of the signalling pathways that directly influence intestinal homeostasis and tumorigenesis. The role of Wnt/β-catenin signalling in both processes was documented by several major discoveries published in the 1990s. In 1996, reports from three laboratories showed that β-catenin, the main mediator of canonical Wnt signalling, interacts with the T-cell factor (TCF)/lymphoid enhancer factor (LEF) and converts these DNA binding proteins to transcriptional activators [1,2,3]. In 1997, several research teams reported that in cancer cells with inactive tumour suppressor adenomatous polyposis coli (APC) or harbouring stabilizing mutations in the *Ctnnb1* gene (the gene encoding β-catenin), TCF/β-catenin-dependent transcription is constitutively active [4,5,6]. In 1998, these results were complemented by gene targeting in the mouse, showing that upon ablation of the β-catenin interacting partner TCF4, proliferative compartments in the small intestine are not formed [7]. In agreement with these observations were data showing that the growth of intestinal organoids depends on Wnt agonists R-Spondins (RSPOs) and organoids derived from APC-deficient intestinal tumours lost this dependency [8,9]. In 2011, de Lau and co-workers documented that the intestinal stem marker leucine-rich-repeat-containing G-protein-coupled receptor 5 (LGR5), whose expression is controlled by Wnt signalling, functions as a transmembrane RSPO receptor [9]. One year later, Koo and colleagues reported that RSPO/LGR signalling potentiates the surface expression of Wnt receptors frizzled (FZD) [10]. These findings seemingly completed our perception of the Wnt pathway as the major regulatory mechanism involved in intestinal epithelium renewal and transformation. The situation changed a few years ago when several laboratories documented that some effects previously attributed to hyperactive Wnt/β-catenin signalling are actually mediated by components of the Hippo pathway [11,12,13]. Additionally, Park and colleagues corroborated an alternative model of Wnt signalling that directly includes some effector proteins regulated by the Hippo pathway [14]. Moreover, several recent articles showed that besides RSPOs, LGR proteins associate with other ligands. Consequently, the mode of the LGR-mediated intracellular response is more complex than originally thought [15,16]. In summary, we attempted to recapitulate the published data related to possible interactions among the Wnt, Hippo and RSPO/LGR pathways. We also summarized some results obtained upon gene inactivation of individual RSPO ligands and LGR receptors in the mouse. 

## 2. Intestinal Epithelium Architecture and Cellular Composition

The most distinct feature of stem cells is their self-renewal capacity and potency, i.e., the ability to differentiate into one or multiple types of differentiated cells. In contrast to embryonic stem cells that populate the early embryo and give rise to the entire organism, adult stem cells emerge during later developmental stages and their self-renewal and cell differentiation potential is limited to a particular organ or tissue [17]. Virtually all adult stem cells are found in a specific tissue microenvironment or compartment called the stem cell niche. The niche has specific physical and (bio)chemical properties and complex cellular composition that supports the stem cell growth. In addition, the niche keeps stem cells in the undifferentiated state, i.e., preserves their *stemness* and also determines stem cell numbers [18].

Intestinal epithelium—similarly to other tissues of the adult organism—is characterized by the presence of stem cells that ensure tissue homeostasis and regeneration upon tissue damage. With constituent cells renewing every 3–5 days, the intestinal epithelium represents the most dynamic cellular system in the mammalian body [19]. In the small intestine, the single-layer epithelium covers microscopic protuberances and invaginations called villi or crypts, respectively. The villi contain differentiated non-dividing cells. In contrast, proliferating cells including intestinal stem cells (ISCs) are localized to the crypts. The discovery indicating the crucial role of the Wnt signalling pathway in small intestinal crypts and ISC homeostasis has significantly influenced the stem cell field in the last twenty years [7]. Moreover, the fact that activation of the Wnt pathway is a typical feature of human intestinal tumours [4,5] enabled efficient combination of experiments using human tumour cells with the methodology utilizing gene targeting in the mouse. 

The idea that gene expression triggered by aberrant Wnt signalling in human intestinal cancer cells mirrors the Wnt-dependent transcription program in the crypt cells led to the discovery of several ISC-specific markers [20,21]. Although the majority of identified Wnt target genes showed a broad expression pattern in the crypt cells, some of the genes displayed more localized expression. Notably mRNA encoding LGR5 was produced in crypt base columnar (CBC) cells, slender, actively dividing cells that were intermixed with Paneth cells at the bottom of the crypt [22]. Subsequent lineage tracing studies revealed asymmetric division of LGR5^+^ cells accompanied by their ability to differentiate into all cell lineages present in the intestinal epithelium. In the small intestine, these cell types include absorptive enterocytes, microfold (M) cells associated with the mucosal immune system and several populations of secretory cells. The latter are represented by mucus-producing goblet cells, various sorts of hormone-secreting enteroendocrine cells, tuft cells that secrete endogenous opioids but also contribute to immune type 2 responses against enteric parasite infections and Paneth cells characteristic by production of anti-bacterial enzymes and peptides [23]. Besides LGR5, additional ISC-specific markers have been identified such as achaete-scute complex homolog 2 (Ascl2), olfactomedin 4 (Olfm4) [24], SPARC-related modulator calcium binding 2 (Smoc2) [25] and Troy (alternative names—tumour necrosis factor receptor superfamily, member 19 (TNFRSF19) or TAJ) [26]. ISCs divide approximately once per day, generating highly proliferative progenitors called transit amplifying (TA) cells, localized in the crypt above the stem cell zone. Dividing cells move upwards from the crypt and, when they reach the crypt orifice, stop to divide and differentiate. Consequently, the differentiated cells on the villus continuously move and, once reaching the villus tip, are shed into the intestinal lumen. One exception from this scheme are Paneth cells. These long-lived cells do not migrate from the crypts but remain at the crypt base for 5–6 weeks. The morphology of the large intestine resembles the small intestine; however, it lacks villi and Paneth cells, the latter being substituted by the recently discovered deep crypt secretory (DCS) cells, marked by their expression of regenerating islet-derived family member 4 (Reg4) [27]. 

Before the stem cell identity of LGR5^+^ CBC cells had been confirmed, a number of studies proposed that intestinal homeostasis is maintained by slowly dividing cells localized four cell positions from the bottom of the crypt [28]. The “+4” cells, due to their quiescence-like state, displayed increased radiation resistance and were capable to repopulate the damaged epithelial layer [29]. However, despite remarkable effort, none of the markers assigned to these “reserved” ISCs proved to be specific [25,30]. However, there is growing evidence related to the high phenotypic plasticity of cells within the intestinal epithelium [31,32]. In the current view, rather than genuine ISCs, the +4 cells are considered to be non-dividing precursors of the secretory cell lineages that under stress conditions can dedifferentiate and replace the damaged or lost ISCs [33].

The ISC niche in the small intestine is composed of the extracellular matrix (ECM) and many cell types that include connective tissues cells (fibroblasts and myofibroblasts), endothelial and immune cells, cells of neuronal origin and smooth muscle and Paneth cells [34]. Interestingly, the tissue niche can be substituted by a “cocktail” of growth factors and signalling ligands that supports growth of isolated crypts or single LGR5^+^ ISCs in culture conditions. Upon embedding in Matrigel, the crypts (or single ISCs) generate three-dimensional structures called organoids or miniguts that resemble the organization, self-renewal and differentiation of the intestinal epithelium. The organoid medium has to be supplemented with the Wnt pathway agonist RSPO, the bone morphogenetic protein (BMP) pathway inhibitor noggin and epidermal growth factor (EGF). Essential factors, additional to RSPO, noggin and EGF, are supposedly produced by ISCs neighbouring Paneth cells. The Paneth cell-derived factors include Wnt3, the Notch ligand delta like 4 (Dll4) and tumour necrosis factor (TNF) [8,35]. Intriguingly, Paneth cell ablation does not impact ISCs in vivo; however, it has a deleterious effect on organoid growth [36]. The organoid cultures can be rescued by intestinal stromal cells, implying redundancy between Paneth cells and stroma as the Wnt source in the organism [37,38].

## 3. Wnt Signalling in the Intestine

Wnt signalling represents one of the key signalling mechanisms that regulates intestinal epithelium homeostasis. Nineteen mammalian secreted Wnt ligands have been identified, which contribute to a multitude of cellular processes such as proliferation, differentiation, cell-fate determination, migration and polarization [39]. Upon translation, all Wnt are acetylated in the endoplasmic reticulum (ER) by the acyl transferase porcupine (PORCN), which catalyses addition of palmitic and palmitoleic acid to the Wnt molecule [40,41]. Acylation is essential for Wnt passing through the secretory pathway and/or signalling properties of the Wnt ligand. Consequently, PORCN inhibition leads to reduction or absence of the Wnt ligand signalling capacity [42,43]. Wnt ligands are classified as canonical (e.g., Wnt1, Wnt3a and Wnt8a) and non-canonical (Wnt4, Wnt5a, Wnt11). Whereas the canonical ligands increase the stability of β-catenin as a key intracellular signalling molecule, non-canonical Wnts act β-catenin independently. The canonical Wnt/β-catenin pathway is triggered by interaction between the (canonical) Wnt ligand and the transmembrane receptor complex that is formed from the seven-pass transmembrane FZD and the co-receptor low-density lipoprotein receptor-related protein 5 or 6 (LRP5/6). Among 10 FZDs encoded in the mouse genome, *Fzd5* and *Fzd7* mRNAs are highly enriched in the crypts and their expression declines towards the villus tip [44]. FZD7 appears to be the crucial Wnt signalling mediator in ISCs, since conditional deletion of the *Fzd7* gene in LGR5^+^ cells leads to stem cells loss and impaired regeneration capacity after damage [45]. Additionally, increased *Fzd7* expression levels were linked to progression of human intestinal cancer [46]. The Wnt-FZD-LRP5/6 interaction prevents activity of the β-catenin destruction complex that is composed of two scaffolding proteins, APC and AXIN and two serine/threonine kinases, casein kinase-1 (CK1) and glycogen synthetase kinase 3 (GSK3). In unstimulated cells, the complex binds β-catenin and sequentially phosphorylates the protein at serine 45 (CK1), threonine 41 (GSK3) and serines 37 and 33 (GSK3). Phosphorylated β-catenin molecules are polyubiquitinated by the E3 ligase β-transduction repeat-containing protein (β-TrCP) and degraded by the proteasome [47]. Upon Wnt stimulation, β-catenin accumulates in the cell cytoplasm and migrates to the nucleus, where it displaces the transcriptional groucho/transducin like enhancer of split (TLE) co-repressors from the nuclear high-mobility group (HMG) box containing DNA-binding factors TCF/LEF family ([48]). Beta-catenin activates transcription by recruitment of transcription co-activators and/or histone modifiers such as cyclic-AMP response element binding protein (CBP), ATP-dependent brahma related gene 1 (BRG1), p300 and B-cell lymphoma (BCL9) [49,50]. 

The contribution of Wnt/β-catenin signalling to intestinal self-renewal and tumorigenesis was established by gene targeting studies performed in the mouse and by analysis of human cancer cells. For example, disruption of the *Tcf7l2* gene, encoding the β-catenin partner and Wnt signalling transcriptional regulator, caused complete loss of the intestinal crypt compartments accompanied by the absence of proliferating cells in the small intestine of new-born *Tcf7l2*^−/−^ mice [7]. A similar phenotype was observed in transgenic animals overexpressing the secreted Wnt signalling inhibitor dickkopf-1 (Dkk1) [51]. In contrast, increased activation of the Wnt pathway represents a hallmark of the majority of intestinal adenomas or adenocarcinomas and the most frequent mutations found in sporadic tumours of the colon and rectum (colorectal cancer (CRC)) are mutational changes inactivating the APC tumour suppressor [52,53,54,55]. Consequently, in APC-deficient cells, the β-catenin destruction complex is not functional and the TCF4-β-catenin-dependent transcription is (aberrantly) activated [4]. In 1990, Moser and colleagues developed a mouse model of intestinal tumorigenesis based on random mutagenesis [56]. Two years later, Su and co-workers identified that the dominant mutation predisposing animals of this multiple intestinal neoplasia (Min) strain to numerous, mainly small intestinal tumours, inactivates the mouse *Apc* orthologue [57]. Correspondingly, conditional inactivation of *Apc* using the Cre/loxP system in LGR5^+^ cells initiates neoplastic growth in epithelia of the small intestine and colon [58]. Another example of genetic changes altering the Wnt pathway components and that have been identified in CRC, are missense mutations in the *Ctnnb1* gene (encodes β-catenin) that stabilize the protein or mutations inactivating the additional component of the β-catenin destruction complex AXIN2 [5,59]. 

The Wnt/β-catenin pathway activity in the intestine generates a descending gradient along the crypt-villus axis that controls epithelial cell division, differentiation and migration. As already indicated, Paneth cells produce Wnt3, which stimulates the neighbouring cells; however, how the Wnt gradient is established and controlled remained unclear [35,44,60]. However, recent data obtained by Farin and colleagues using an epitope-tagged, functional *Wnt3* knock-in allele, brought valuables insights into the Wnt ligand movement in the intestine. The ligand is distributed in a single-cell distance from its source (Paneth cell) to the neighbouring ISC. Then, the Wnt-FZD complex is passively distributed to daughter cells through mitosis. Subsequent cell divisions in the ISC and TA compartments results in dilution of surface-bound Wnt leading to the gradient formation [61]. 

The less-well characterized non-canonical Wnt pathways include Wnt/planar cell polarity (Wnt/PCP) and Wnt/calcium (Wnt/Ca^2+^) signalling. In Wnt/PCP signalling, the Wnt signal is transduced by a transmembrane receptor complex that involves the FZD receptor and the alternative co-receptors receptor tyrosine kinase-like orphan receptor 1/2 (ROR1/2), receptor-like tyrosine kinase (RYK) and protein tyrosine kinase 7 (PTK7). Wnt/PCP signalling activates the small GTPases RHO/RAS-related C3 botulinum toxin substrate 1 (RAC1) and results in cytoskeleton reorganization affecting cellular polarity and migration [62]. In Wnt/Ca^2+^ signalling, the Wnt FZD interaction leads to activation of phospholipase C (PLC) through heterotrimeric G proteins. Active PLC hydrolyses phospholipids to inositol 1,4,5-trisphosphate (IP3) and 1,2-diacyl glycerol. These molecules function as “second messengers” that induce multiple cellular responses such as Ca^2+^ release from the ER and protein kinase C (PKC) stimulation [63]. The role of non-canonical pathways in intestinal homeostasis has not been clarified yet. Wnt/PCP signalling possibly antagonizes the canonical Wnt/β-catenin pathway. Accordingly, increased production of Wnt5a is associated with improved prognosis in patients with intestinal tumours [64,65]. In contrast, Bakker and colleagues reported the opposite, i.e., correlation between Wnt5a expression and invasiveness of human and mouse colon cancer cells [66]. Thus, the exact role(s) of non-canonical pathways in intestinal homeostasis or tumour formation remains to be clarified.

## 4. R-Spondins/Leucine-Rich-Repeat-Containing G-Protein-Coupled Receptor Signalling

**Figure 1:**
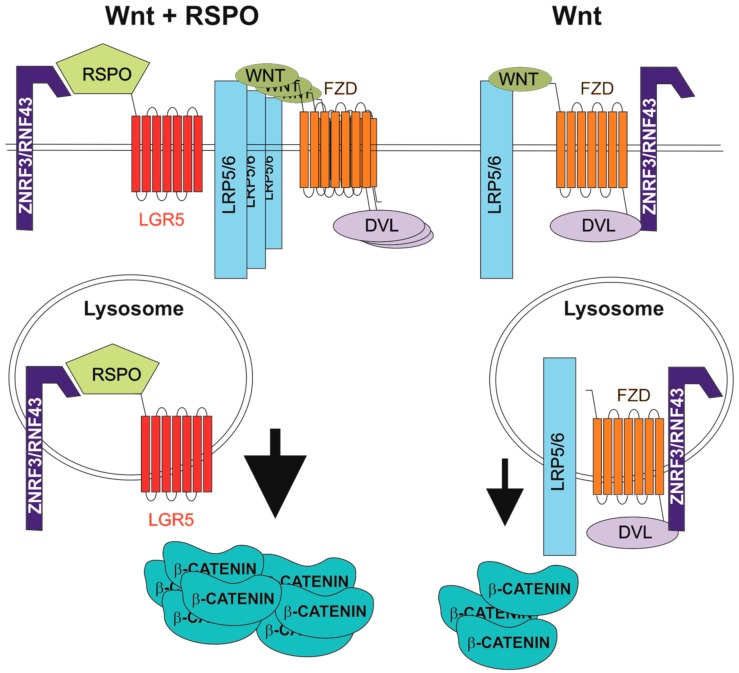
Enhancement of Wnt signalling by R-spondin (RSPO) ligands. DVL, dishevelled; FZD, frizzled; LGR5, leucine-rich-repeat-containing G-protein-coupled receptor 5; LRP5/6, low-density lipoprotein receptor-related protein 5 or 6; RNF43, ring finger 43; ZNRF3, zinc and ring finger 3.

The human genome harbours four genes that encode related RSPO proteins. All RSPOs include a signal peptide, two furin-like cysteine-rich domains (FU1-CRD and FU2-CRD), a thrombospondin type 1 repeat domain and a basic amino acid-rich repeat domain [67]. Based on the physical interaction between RSPO1 and LRP6, RSPOs were first described as Wnt signalling agonists [68]. In 2011, three laboratories reported that RSPOs bind LGR5 (and its homologs LGR4 and LGR6) and that this interaction enhances the Wnt pathway output [9,69,70,71]. In the present model, RSPO-mediated potentiation of Wnt signalling depends on the cell surface clearance of the homologous transmembrane E3 ubiquitin ligases, ring finger 43 (RNF43) and zinc and ring finger 3 (ZNRF3). The ligases antagonize both canonical and non-canonical Wnt signalling through ubiquitination and degradation of Wnt receptors. In the presence of RSPO, a specific RSPO-LGR-RNF43/ZNRF3 complex is formed and the RNF43/ZNRF3 activity is suppressed [72]. Mechanistically, RNF43/ZNRF3 and LGR interact with the RSPO FU1-CRD and FU2-CRD domains, respectively. This leads to internalization of the ligases and their lysosomal degradation [73,74] (Figure 1). Recently, Loregger and co-workers determined another mode of RNF43 inhibitory action on Wnt signalling. RNF43 interacts with TCF4 and tethers the protein to the nuclear membrane. Since RNF43 functions even in cells with constitutive β-catenin activity, mimicking (or potentiation) of this specific RNF43 function might be used to treat CRC initiated by aberrant Wnt signalling [75]. However, based on an RNF43/ZNRF3 knockdown study, Carmon and colleagues suggested an alternative E3 ubiquitin ligase-independent, mechanism underlying the enhancement of Wnt pathway activity by RSPO-LGR signalling. RSPO1-LGR4 interacts with IQ motif containing GTPase activating protein 1 (IQGAP1) and the supramolecular complex recruits the cytoplasmic mediator of Wnt signalling, dishevelled (DVL). The complex promotes LRP5/6 phosphorylation, leading to increased canonical Wnt signalling [76]. This finding was later questioned by Jiang and colleagues, who analysed the results upon Clustered Regularly Interspaced Short Palindromic Repeats (CRISPR)/Cas9-mediated ZNRF3/RNF43 knockout in human cells. These authors highlighted the interaction between RSPO and RNF43/ZNRF3 as the main mechanism related to the “boost” of Wnt signalling. Moreover, they proposed a dual role for DVL in Wnt signalling regulation. In the absence of Wnt, DVL mediates interaction with FZD and ZNRF3/RNF43 and promotes receptor internalization. Upon ligand engagement, FZD is released from the interaction with DVL and RNF43/ZNRF3 stabilizing receptor expression on the cell surface [77].

In the intestine, RSPO1/2/3 mRNAs are predominantly produced in the stroma, whereas their expression in the epithelium is negligible [38]. The expression pattern corroborates the necessity to supplement organoid culture medium with exogenous RSPO to support organoid growth [8]. Interestingly, mice with conditional deletion of the *Porcn* gene in the intestinal epithelium showed no pathological changes but organoids derived from these mice failed to grow. Additionally, organoid viability was rescued by co-culture with cells producing RSPO1 (and Wnt3a). These findings support the idea that stromal production of RSPOs and Wnts is sufficient to support intestinal homeostasis [38]. Very recently, two articles brought an important insight related to Wnt and RSPO function in ISC self-renewal. The authors generated water-soluble Wnt agonists heterodimerizing the FZD–LRP5/LRP6 molecules and used them to activate Wnt signalling in experimental mice. Additionally, RSPOs were supplied as recombinant adenoviruses encoding RSPO1/2 (Ad-RSPO1/2). Moreover, these authors also achieved in vivo inhibition of Wnt/RSPO-mediated signalling by using adenoviral viruses producing FZD-CRD, Dkk1 (Ad-FZD-CRD, Ad-Dkk1), or RNF43/ZNRF3 and LGR5 soluble ectodomains (Ad-RNF43/ZNRF3-ECDs, Ad-LGR5-ECD). Intriguingly, contrary to the studies published previously [78], stimulation of the Wnt receptor did not induce crypt hyperplasia or increased ISC numbers. In contrast, infection with Ad-RSPOs provoked crypt (hyper)proliferation and increased production of LGR5^+^ cells. As expected, Wnt pathway inhibition (infection with Ad-FZD-CRD/Ad-Dkk1) caused crypt demise that was not rescued by RSPOs. Similarly, crypts were damaged upon RSPO signalling inhibition (upon infection with Ad-RNF43/ZNRF3-ECDs and Ad-LGR5-ECD); however, in this particular case the crypt formation was rescued by (simultaneous) Wnt signalling activation. In conclusion, these studies underlined the role of Wnt ligands in priming epithelial cell renewal via establishment of the ISC fate. RSPO signalling can induce LGR5^+^ cell amplification; however, the signalling is insufficient for formation (or maintenance) of ISCs when the Wnt signal is absent [79,80].

LGR5 and related LGR4 and LGR6 proteins represent the B subgroup of LGR transmembrane proteins, related to the G-protein-coupled receptors (GPCRs) of the hormone receptor class. All LGRs contain a large N-terminal extracellular domain; however, in contrast to LGR4/5/6, the other members of the LGR A and C subgroups bind hormones [81,82,83]. LGR4/5/6 proteins are highly homologous; nevertheless, their expression pattern appears to be only partially overlapping. As already described, LGR5 was identified as a Wnt signalling-regulated marker of ISCs [22]. Additionally, subsequent studies established LGR5 as a marker of stem cells in the hair follicle, stomach, mammary gland and many other mouse tissues [84,85,86,87,88,89]. Rather surprisingly, *Lgr5* knockout mice develop normally but die shortly after birth because of ankyloglossia, a craniofacial defect that prevents new-borns from suckling. Otherwise, LGR5-deficient animals do not display any (remarkable) phenotypic abnormalities [90]. The other subgroup member, *Lgr6*, is expressed in the embryonic hair placode and in the area interconnecting adult hair follicles with sebaceous glands. The LGR6^+^ cells were identified as adult stem cells with the potential to generate all skin cell lineages. Remarkably, *Lgr6* expression is Wnt signalling independent and *Lgr6* knockouts do not manifest any apparent defects [91]. In contrast, LGR4 is expressed in proliferating cell compartments of many tissues including the bone, gastrointestinal tract, kidney, liver, mammary gland, pancreas and skin. Consequently, whole-body disruption of the *Lgr4* gene is embryonic/perinatal lethal [92,93]. In the mouse crypt, conditional ablation of *Lgr4* had damaging effects on ISC proliferation. Deficiency in both *Lgr4* and *Lgr5* genes worsened the phenotype and led to crypt demise [9]. *Lgr4* gene expression is activated by BMP2 in osteoblastic cells [94] or by signal transducer and activator of transcription 3 (STAT3) signalling in osteosarcoma cells [95]. The latter observation is in agreement with the phenotype of *Lgr4* null mice, which display delayed osteoblast differentiation [96]. It also corroborates the finding that the Wnt pathway regulates bone mass, skeletogenesis and maintenance of adult skeleton [97]. Interestingly, Luo and colleagues discovered that LGR4 functions as an alternative receptor for RANKL (also known as TNFSF11). In canonical signalling, RANKL binds its “classical” receptor RANK, inducing differentiation of osteoclasts and thereby bone resorption. In pathological conditions, the signalling might lead to osteoporosis [98]. In contrast, LGR4 produced on osteoclasts acts in the opposite manner and the RANKL-LGR4 pathway blocks osteoclastogenesis and bone resorption [16]. In agreement with these results, whole-genome sequencing and single-nucleotide polymorphism (SNP) chip analysis of more than 64 thousand Icelanders revealed the presence of a rare nonsense, i.e., inactivating, mutation in the *Lgr4* gene that is linked to low bone mineral density [99]. Interestingly, a different gain-of-function LGR4 variant is linked with central obesity and a metabolic phenotype in young Chinese [100]. Complementary to this finding, ablation of mouse *Lgr4* stimulates white-to-brown fat transition and promotes energy expenditure [101]. Currently, Deng and colleagues revealed that the BMP2 antagonist norrin interacts with LGR4 and that norrin-LGR4 potentiates the Wnt pathway similarly as the RSPO-LGR4 interaction [102]. Finally, Planas-Paz and colleagues showed that in the mouse liver, RSPO1-LGR4 signalling controls organ size and tissue regeneration. Nevertheless, the authors show that some of the effects mediated by RSPO1-LGR4 signalling are Wnt pathway independent [15]. In summary, all these results imply that the role of LGR4-mediated signalling is not limited to intestinal tissue and in addition, the receptor is possibly involved in multiple intracellular signalling mechanisms. 

Similar to LGRs, RSPO functions do not (completely) overlap. Nevertheless, all RSPOs have the capacity to induce crypt cell proliferation and β-catenin activation [103]. RSPO1-deficient mice are viable; however, RSPO1 absence affects their ovary development [104]. *Rspo2* gene knockout is associated with facial skeletal defects, limb loss and lung hyperplasia resulting in perinatal respiratory failure [105]. RSPO3 deficiency results in embryonic lethality, due to aberrant placental development and cardiovascular abnormalities [106,107]. Results related to *Rspo4* gene targeting are not available yet; however, in humans, mutations affecting RSPO4 function are associated with anonychia, the absence of finger- and toenails [108].

Aberrant RSPO/LGR signalling is frequently associated with CRC. Interestingly, in a fraction of CRCs harbouring intact *Apc*, *Ctnnb1* or *Axin2* genes, genetic rearrangements in the *Rspo2* or *Rpso3* locus were identified. The detected alterations generate fusions between *Rspo2* and the eukaryotic translation initiation factor 3, subunit E (*Eif3e*) gene or between *Rspo3* and the protein tyrosine phosphatase, receptor type, K (*Ptprk*) gene. Both fusions possibly lead to increased production of functional RSPO proteins resulting in aberrant Wnt signalling [109,110]. Furthermore, upregulation of LGR5 in colorectal tumours was associated with poor patient prognosis. This latter phenomenon is possibly associated with the involvement of LGR5^+^ tumour cells in relapse after therapy [111,112]. No direct association between LGR4 and CRC has yet been reported; however, increased LGR4 mRNA levels were observed in prostatic cancer [113]. Nevertheless, mutations inactivating LGR4 were associated with an increased incidence of biliary tract, gallbladder and skin cancer, suggesting a tumour suppressor role of LGR4 [99].

## 5. The Hippo Pathway

The Hippo signalling pathway was recently added to the list of signalling mechanisms involved in intestinal tumorigenesis. Importantly, several of the Hippo and Wnt pathway components are either shared between both pathways, or physically interact. The Hippo pathway was originally discovered in *Drosophila melanogaster* as an organ size regulatory system; however, its core components are evolutionally conserved [114,115]. The main modules identified in the mammalian Hippo pathway include serine/threonine STE20-like protein kinase 1 (MST1; alternative name STK4) and related MST2 (STK3), which are homologous to Drosophila Hippo, large tumour suppressor kinase 1/2 (LATS1/2) and scaffold proteins salvador family WW domain containing protein 1 (SAV1) and MOB Kinase Activator 1A/1B (MOB1A/1B). The pathway is stimulated when the cell receives growth inhibitory signals upon sensing high cell density and contact inhibition. The pathway regulates cellular localization of two proteins, Yes-associated protein (YAP1) and tafazzin (TAZ) [116,117]. Upon MST1/2 activation—via an extracellular stimulus and transmembrane signal relay mechanism that have not been clarified yet—MST1/2 form a complex with SAV1. Subsequently, the signal activates kinase LATS1/2 that interacts with MOB1A/1B. LATS1/2 phosphorylates YAP1 at serine 127 [118,119] and TAZ at serine 89 [120]. The phosphorylation enables binding of YAP1/TAZ to 14-3-3 proteins and prevents their transport to the nucleus. LATS-mediated phosphorylation of YAP1 at serine 381 primes subsequent phosphorylation at serine 384 and serine 387 executed by CK1. Phosphorylated YAP1 is recognized by β-TrCP, which leads to YAP ubiquitination and degradation [121].

In the Hippo signalling *OFF* state, YAP1 and TAZ mediators are translocated into the nucleus, where they bind members of the TEA domain transcription factor (TEAD) family [122] and promote transcription of pro-proliferative and anti-apoptotic genes. Alternatively, YAP1 and TAZ associate with runt-related transcription factor 2 (RUNX2) [123], SMAD [124] and AP-1 transcription complexes [125]. YAP1/TAZ can also function as transcription co-repressor after recruiting the nucleosome remodelling and deacetylase (NuRD) complex [126]. The absence of cell-cell contacts leads to RHO activation that, together with the actin cytoskeleton, promotes YAP1/TAZ nuclear translocation. This implies that cell contacts (or cell adhesion to the extracellular matrix) might be the impulse activating Hippo signalling. Another mechanism affecting YAP1/TAZ nuclear translocation is mediated by GPCRs that act via RHO-dependent LATS1/2 inactivation and YAP1/TAZ nuclear translocation [14]. 

**Figure 2:**
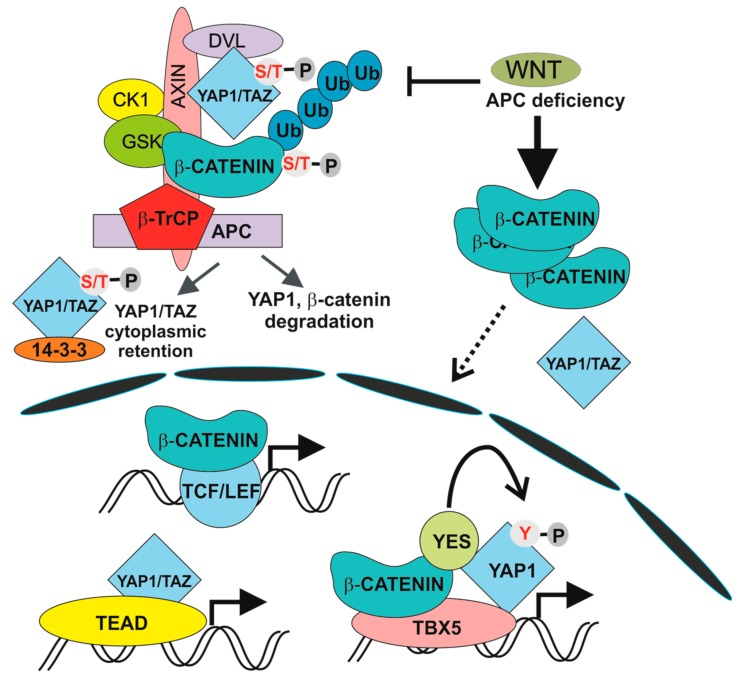
Transcription complexes induced by physiological and aberrant Wnt pathway activation. β-TrCP, β-transduction repeat-containing protein; APC, adenomatous polyposis coli, CK1, casein kinase-1, GSK3, glycogen synthetase kinase 3; S/T-P, the phosphorylated serine or threonine residue; TAZ, tafazzin; TEAD, TEA domain transcription factors; TBX5, T-box 5; TCF/LEF, T-cell factor /lymphoid enhancer factor; Ub, ubiquitin; Y-P, the phosphorylated tyrosine residue; YAP1, Yes-associated protein; YES; YES proto-oncogene 1.

**Figure 3:**
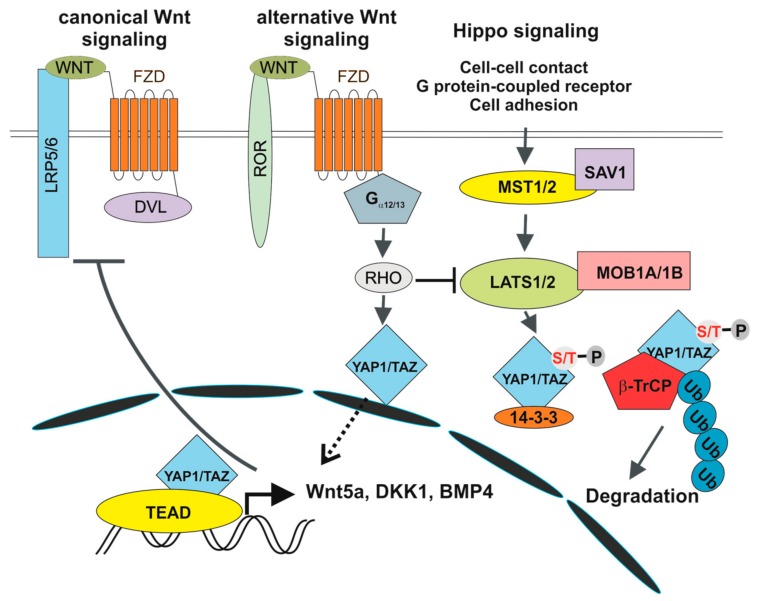
Crosstalk between canonical and alternative Wnt signalling and the Hippo pathway. 14-3-3, 14-3-3 protein; BMP4, bone morphogenetic protein 4; DKK1, dickkopf-1; Gα_12/13_, G12/G13 alpha subunits of heterotrimeric G proteins; LATS1/2, large tumour suppressor kinase 1/2; MOB1A/1B, MOB Kinase Activator 1A/1B; MST1, STE20-like protein kinase 1; RHO, RHO GTPase; ROR1/2, receptor tyrosine kinase-like orphan receptor 1/2; SAV1, salvador family WW domain containing protein 1.

The involvement of the Hippo pathway in intestinal tumorigenesis was revealed by Camargo and co-workers. They showed that inducible YAP1 (over)expression in the mouse induced intestinal hyperplasia accompanied by the absence of mature Paneth and goblet cells. The observed phenotype, which was reversible and strictly dependent on YAP1 production, was not caused by cell dedifferentiation. Instead, differentiated cells were replaced by newly arising YAP1-expressing progenitors [11]. In addition, several studies associated deregulation of the Hippo pathway with poor prognosis of CRC patients [127,128]. There is growing evidence for multiple crosstalk between Hippo and Wnt/β-catenin signalling, although some results are controversial. Zhou and colleagues reported stimulation of Wnt/β-catenin signalling upon inactivation of MST1/2 kinases [128]. In addition, co-immunoprecipitation studies showed interaction between DVL and TAZ. Moreover, TAZ inhibited Wnt3a-induced DVL phosphorylation [129]. Similarly, Barry and colleagues reported functional interaction between YAP1 and DVL. YAP1 was detected in the cytoplasm of TA cells and in epithelial cells present on the villi, where it attenuated Wnt signalling by inhibiting DVL function. Moreover, overexpression of YAP1 mutant localized to the cytoplasm blocked crypt proliferation and induced intestinal degeneration. Additionally, YAP1 absence caused Wnt hypersensitivity during tissue regeneration that was connected with intestinal stem cell niche expansion. In their interpretation, the authors conclude that DVL nuclear translocation is the rescue mechanism in response to intestinal injury and that YAP1 controls this localization [130]. Additionally, direct interaction between β-catenin and phosphorylated YAP1/TAZ was reported to retain β-catenin cytoplasmic localization [131]. Intriguingly, Cai and colleagues documented interaction between SAV1, LATS and APC independently of β-catenin destruction complex formation. Consequently, APC deficiency leads to transcription of YAP1-specific target genes, including TAZ and β-catenin. In addition, ablation of the *Yap1* or *Taz* gene abolished intestinal adenoma formation in APC-Min mice [132]. In contrast, the Piccolo group demonstrated direct regulation of TAZ and YAP1 activity by the β-catenin destruction complex. Upon Wnt pathway activation (and β-catenin destruction complex inactivation) YAP1 and TAZ are stabilized, translocate to the nucleus and together with TEAD transcription factors, drive expression of a specific set of target genes (Figure 2). The authors propose that a substantial portion of the genes regulated by aberrant Wnt signalling in CRC cells are in fact activated by YAP1/TAZ-TEAD complexes. The conclusion was supported by an intriguing observation showing that tumour formation in the intestine induced by conditional ablation of the *Apc* gene is completely suppressed by simultaneous inactivation of *Yap1* and *Taz* [12,13]. Interestingly, individual and double deficiency of YAP1 and TAZ had no effect on crypt cell proliferation and intestinal architecture, indicating that YAP1/TAZ are dispensable for intestinal homeostasis [12]. A different model explaining the dependency of intestinal tumorigenesis on YAP1 was presented by Rosenbluh and colleagues. The authors performed a genome-scale loss-of-function screen to identify genes essential for cancer cells displaying active Wnt/β-catenin signalling. They found that proliferation and cell survival of the majority of CRC cells depends on the transcriptional activity of a protein complex that involves β-catenin, YAP1 and T-box 5 (TBX5) DNA-binding protein [133]. Interestingly, β-catenin transcriptional activity is influenced by the methylation status of YAP1. YAP1 methylated at lysine 494 by SET domain containing lysine methyltransferase 7 (SETD7) binds β-catenin and promotes its nuclear localization and transcription of Wnt target genes [134]. Besides that, YAP1 phosphorylation by YES proto-oncogene 1 tyrosine kinase (YES1) impacts on YAP1 transcriptional activity in CRC cells [133] (Figure 2). Finally, Park and colleagues corroborated an alternative model of Wnt signalling that directly includes some of the Hippo pathway components. In the “alternative” Wnt signalling pathway, the engagement of both types of canonical and noncanonical Wnt ligands with the receptor complex composed of FZD and ROR triggers a cascade of intracellular events that includes G12/G13 alpha subunits of heterotrimeric G proteins (Gα_12/13_), RHO GTPases, and LATS1/2 promoting YAP1/TAZ activation and TEAD-mediated transcription (Figure 3). In primary human bone-marrow stem cells, the pathway regulates osteogenic differentiation and cell migration. Intriguingly, some of the genes activated by the pathway encode inhibitors of the canonical Wnt pathway (e.g., DKK1) [14]. However, whether alternative Wnt signalling is a common signalling mechanism or is restricted to some specific cell types has yet to be defined.
